# People solve rebuses unwittingly—Both forward and backward: Empirical evidence for the mental effectiveness of the signifier

**DOI:** 10.3389/fnhum.2022.965183

**Published:** 2023-02-10

**Authors:** Giulia Olyff, Ariane Bazan

**Affiliations:** ^1^Centre de Recherche en Psychologie Clinique, Psychopathologie et Psychosomatique, Université libre de Bruxelles (ULB), Bruxelles, Belgium; ^2^Observatoire du SIDA et des Sexualités, Université libre de Bruxelles (ULB), Bruxelles, Belgium; ^3^Interpsy (UR 4432), Université de Lorraine, Nancy, France

**Keywords:** rebus, signifier, Freud, Lacan, phonological priming, Shevrin

## Abstract

**Introduction:**

Freud proposed that names of clinically salient objects or situations, such as for example a beetle (*Käfer*) in Mr. E’s panic attack, refer through their phonological word form, and not through their meaning, to etiologically important events—here, “*Que faire?*” which summarizes the indecisiveness of Mr. E’s mother concerning her marriage with Mr. E’s father. Lacan formalized these ideas, attributing full-fledged mental effectiveness to the signifier, and summarized this as “the unconscious structured as a language”. We tested one aspect of this theory, namely that there is an influence of the ambiguous phonological translation of the world upon our mental processing without us being aware of this influence.

**Methods:**

For this, we used a rebus priming paradigm, including 14 French rebuses, composed of two images depicting common objects, such as *paon* /pã/ “peacock” and *terre* /tεr/ “earth,” together forming the rebus *panthère* /pãtεr/ “panther.” These images were followed by a target word semantically related to the rebus resolution, e.g., *félin* “feline,” upon which the participants, unaware of the rebus principle, produced 6 written associations. A total of 1,458 participants were randomly assigned either to Experiment 1 in which they were shown the rebus images in either forward or in reverse order or to Experiment 2, in which they were shown only one of both rebus images, either the first or the last.

**Results and discussion:**

The results show that the images induced inadvertent rebus priming in naïve participants. In other words, our results show that people solve rebuses unwittingly independent of stimulus order, thereby constituting empirical evidence for the mental effectiveness of the signifier.

*In fond memory of Howard Shevrin (1926-2018)*

*careful reader of Freud and revolutionary scientist of the human mind.*

## 1. Introduction

Lacan (1966, p. 868) proposed that “the unconscious is structured as a language” and in previous theoretical work, elaborated in “*Phantoms in the voice. A neuropychoanalytic hypothesis on the structure of the unconscious*” (Bazan, 2007), the second author spelled out an interdisciplinary neuropsychoanalytic framework for this claim. This research now proposes an experimental testing for one aspect of Lacan’s proposal, namely that the world is also apprehended as a phonological trace, independently of its semantic meaning. To show this, we have devised a supraliminal rebus priming paradigm in which two images were presented side by side (e.g., an image of *paon* /pã/ “peacock” and of the *terre* /tεr/ “earth”), which together form a new French word or rebus (namely *panthère* /pãtεr/ “panther”). After looking to the images for 4s, the participants were asked to write their first 6 associations to a target word, semantically related to the rebus resolution (e.g., *félin* “feline”). If there were more rebus resolution words (in the given example, *panthère*) in these associations than there were in a control priming, in participants who stayed naive to the protocol, then it was considered that people can solve rebuses unwittingly, thereby constituting evidence going into the direction of Lacan’s proposition.

In the fifties of the previous century, Lacan (1955) operates what is called a “return to Freud”. One of his major concerns in doing so, is that scholars after Freud read his texts on the semantic interpretational level. Indeed, Freud’s major interpretation myths, such as the Oedipus and the Narcissus myth, the Myth of Primitive Hord, and the castration anxiety, are in a way so mind-blowing that they easily take up the whole mental space. We are left blinded by these storylines with echoes all through mythology, literature, and the arts, and thereby, we tend to overread or neglect another major discovery. Freud indeed reveals another shocking new take on how humans grasp the world. First, we must acknowledge that the reigning cognitive paradigm for the way in which humans grasp the world is overwhelmingly visual-semantic (see e.g., Kosslyn, 1994): this is, humans tend to understand the world—the external as well as the internal imagery world—on the semantic interpretation of its imagery appearance. Freud, however, understands that another logic, a phonological one, also underlies our mental grasping of things. In a letter to his friend Wilhelm Fliess, on 29 December 29 1897 (Freud, 1897), he describes the case of Mr. E. This patient evokes his panic attacks as a ten-year-old when trying to catch a black beetle, a *Käfer* in German. It is Mr. E himself who in the session reveals the mental meaning of this black beetle, by shifting the phonological reading from *Käfer* to the French *Que faire?* which, when pronounced with a German accent, sounds about the same way (Mr. E in fact learned French before learning German with his French nanny). However, *Que faire?* means “What to do now?”, a key phrase reflecting both Mr. E’s central symptom—his indecisiveness—and one of the probable etiological origins of his distress, namely the inability of his mother to make up her mind concerning her marriage—which concerns, of course, the choice for Mr. E’s father. At the end of this letter, Freud adds the Yiddish expression *Meschugge!* approximately meaning “isn’t this completely crazy?”. But as a true scientist, Freud does not discard the parts of reality which do not please him or which do not work with his (rational) vision of the human condition. Instead, he starts to gather more materials of the phonological logic underlying mental symptoms, such as everyday life accidents (the forgetting of words, e.g., *Signorelli* in *Psychopathology of Everyday life*; Freud, 1901) and obsessions (e.g., the rat-obsession in *The Ratman*; Freud, 1909). He even notices it in non-pathological preferences: “a young man had exalted a certain sort of ‘shine on the nose’ into a fetishistic precondition. The surprising explanation of this was that the patient had been brought up in an English nursery but had later come to Germany, where he forgot his mother-tongue almost completely. The fetish, which originated from his earliest childhood, had to be understood in English, not German. The ‘shine on the nose’ [in German ‘*Glanz auf der Nase*’] was in reality a ‘*glance* at the nose.’ The nose was thus the fetish, which, incidentally, he endowed at will with the luminous shine which was not perceptible to others.” (Freud, 1927/1931; p. 152) and in disgusts: “A young lady can’t retrieve the name of the Lewis Wallace novel ‘Ben Hur’. When she analyses this forgetting, she realizes in the aftermath that it is because of its phonological closeness to *bin Hure* ‘I am a whore’ which, as she says: « contains an expression that I do not care to use, especially in the company of young man” (Freud, 1901, p. 41). Mostly such a rebus structure of our ‘symptoms’ remains obscure, even to ourselves, but in some personality structures the unconscious is at the surface and rebus reading is mobilized consciously. What is common to all these symptomatic expressions is that phonological ambiguity is mobilized (unconsciously or consciously) to operate drastic shifts in semantic realms, sometimes directly expressed in behavior (e.g., the preference for a shine on the nose), obscuring the mediating role of language. There are many other examples throughout Freud’s whole *oeuvre*, in the first place *in The Interpretation of Dreams* (Freud, 1900).

When it comes to dreams, Freud (1900, p. 242), is indeed elated when he says: “… a dream is [such] a picture-puzzle,^1^ and our predecessors in the art of dream-interpretation have made the mistake of judging the rebus as an artistic composition. As such, of course, it appears nonsensical and worthless.” Several examples of such rebus readings in dreams were given before (Steinig et al., 2017, pp. 2–3). To repeat a straightforward one, in an example of the second author’s practice, a woman, upon coming back from South Africa, dreamt she was paying in a bar with white pieces of paper upon which a big square was drawn almost filling the square. The figurative value of this dream leaves us clueless. But, when she recounts the dream, she indicates she was paying with pieces of paper on which “rand”-s were drawn, *rand* being both the Dutch word for edge and for the South African currency: the rebus reading reveals the meaning. In a new example, a patient dreamt that people were running 78 times back-and forwards on a sand beach, and this dream made sense with the simple intervention “78 rotations per minutes,” or 78 RPM—the dreamer thereupon remembering an emotional childhood scene with a DJ playing records on a vacation beach. Judging the dream “as an artistic composition,” i.e., on the face validity of the image, would have left us stuck on the back-and forth running with a mysterious 78—possibly leading to mystical interpretations of the “magical 7” followed by the “eternal 8.” The rebus interpretation, which arises from the way in which the analysand addresses the dream, on the other hand, gives access to one precise memory. In other words, it is when we read the dream as a picture-puzzle or a rebus, that it delivers (some of) its underlying secrets.

Common to these diverse examples is thus the observation that it is through the phonological structure of the involved name—and not primarily through the semantic signification of the involved “thing”—that key element in symptoms refer to the subject’s history and suggest a plausible rationale for the symptom or the inclination of the subject. Freud even explains how such a “background” network of singular intensities around phoneme groups influences our word choices unconsciously when we speak: “We think we are generally free to choose words and images to express our ideas. But a closer observation shows that it is often considerations extraneous to the ideas that decide this choice and that the form in which we mold our ideas often reveals a deeper meaning, which we do not realize ourselves. (…) some of these images and ways of speaking are often allusions to subjects which, while remaining *in the background*, exert a powerful influence on the speaker. I know someone who, at one time, continuously used, (…) the following expression: ‘When something suddenly crosses the head of someone.’ Now I knew that the man who spoke in this way had recently received the news that a Russian projectile had passed through the field-cap which his son, a fighting soldier, had on his head.” (Freud, 1901, p. 239; our translation and our Italics) and we have proposed a neuroscientific explanation for these observations (Bazan, 2007, 2011; Bazan et al., 2019).

It might seem quite a challenge to find a way to put this proposition to the test and we do not pretend we propose here its full operational equivalent. However, the phonological symptom logic, shown above, supposes that the surrounding world, even when we do not specifically convene it linguistically, has nevertheless a mental influence upon us through its specifically linguistic structure. Importantly, the specifically linguistic influence can only be revealed as a ‘phonological’ influence: indeed, if an exclusively semantic influence was shown, it might be disputed that is should obligatorily be a linguistic one, as it might have been understood as an experiential or imagery influence, translated secondarily in words. But for example, the black beetle is supposed to induce anxiety upon Mr. E. not solely through the black, erratic appearance of its “thing,” the animal beetle, but (importantly) through the linguistic structure of its name, *Käfer*, referring to the anxiogenic ambivalent choice of Mr. E’s mother for Mr. E’s father: *Que faire?* (“What to do now?”). But also, the shiny noses of women are supposed to induce excitement upon the young English-German patient through the phonological equivalence between *Glanz* and glance and the Ben Hur book title is thought to induce aversion through the phonological closeness with *bin Hure*. Here is another example entrusted to the second author: “*Amici* is the name of a shop I once went to with my parents when I was thirteen. I never forgot the name and I also really wanted a coat from that store. Once in India it suddenly turned out that our old caretaker of the orphanage was called ‘*Amaci*.’ That was her nickname and means ‘mother.’ We were adopted at 11 months”. Here, the shop, in a rebus manner, translates to the mothering caretaker, affecting the mental realm of the teller. In other words, even when we do not consciously speak the world, an unconscious influence of its phonological translation upon our mental processing is supposed.

It is therefore disconcerting to us, as it was to Lacan (1955), to observe that the structural importance of language in organizing unconscious mental life, is largely dismissed in modern psychoanalysis. Though in our reading, Freud does his utmost best, on almost every page of the *Interpretation of dreams* (1901) for example, to underscore the formally linguistic weave of dreams, it comes to us that modern psychoanalysis and neuropsychoanalysis, classify (away) linguistic phenomena as conscious, secondary, even cognitive, processes and do not consider linguistic structure as constitutive of unconscious mental life, despite the many examples all through Freud’s writings. For example, we deplore the way “signifiers” or “word presentations” are sometimes classified among other non-linguistic “signifiers” in modern psychoanalysis. This, to us, is a more subtle way of doing away with the radical consequences of the constitutive role of signifiers, namely that our lives and “personalities” are also influenced by coincidental forms which do not follow a semantic, rational logic but operate upon all of us in an illogical, crazy—*Meschugge!*—way. Whatever other motor species one would wish to classify under “signifiers,” there is no motor sequence of any other kind which can switch radically from semantic realm while remaining mechanically completely identical, i.e., in the precise physiology and biomechanics of the involved muscles and joints: this is the unique characteristic of language and this has consequences for our mental constitution, as shown in the clinical examples.

**FIGURE 1:**
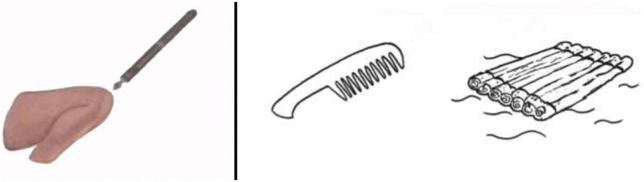
Rebuses from Shevrin and Luborsky (1961) and from Steinig et al. (2017). **(Left)** The *penny* rebus composed of the image of a pen and of a knee from the Shevrin and Luborsky (1961) experiment; **(right)** the rebus *kampflos* (without a fight) composed of the image a comb (*Kamm*) and of a raft (*Floß*) from the Steinig et al. (2017) experiment.

It is also this aspect—the influence of the phonological translation of the world upon our mental processing—that we are putting to the test in this research. For doing this, we are using a rebus paradigm. Indeed, in a rebus the image does not replace a word but the sound—or even better the *phonology* or articulation (Bazan, 2007)—of that word (Préaud, 2004). A rebus here is constituted of two images denoting things, which, translated into their names and put side by side, form a new word, with a radically different meaning. For example, Shevrin and Luborsky (1961)’s most well-known rebus in their early dream-rebus studies is composed of the image of a pen, followed by the image of knee (see also Figure 1), resulting in the rebus ‘penny’. When these images were flashed for 6 ms before sleeping, this led to more ‘penny’ associates (e.g., coin, money) than the control rebus when people were awakened in their REM phase of dreaming. This research was recently replicated by the German researcher Steinig et al. (2017) with the *kampflos* rebus—meaning “without a fight” in German—formed by the image of a comb (*Kamm*)— and of a raft (*Floß*; see Figure 1), leading to essentially the same results.

What this research shows and what ours propose to test is the general “rebus-functioning” mode of the human mental assessment of the world, as illustrated by the clinical examples, more than the empirical confirmation of a clinically observed rebus-resolution instance. Indeed, it is noteworthy that even if “the dream is a rebus” is a key phrase in Freud’s oeuvre, we have found no example of a real rebus or word-puzzle, involving the combination of the names of two or more pictorially represented objects, in the whole of Freud’s writings. As shown, there are many examples involving the necessity to translate an object, a disposition, or an action into language, to obtain the key to a dream or a symptom, but none of them involving a combination of several juxtaposed images—except in Freud’s (1900, p. 242) theoretical example: “a house with a boat on its roof, a single letter of the alphabet, the figure of a running man whose head has been conjured away,” even if, frustratingly, he does not give the resolution of this rebus. Of course, it is fair to suppose that rebus resolution as the phonological translation of two or more juxtaposed images indeed as such also happens in dreams and symptomatic formations, but is less easy to detect, to start by the subject him or herself. That this supposition is not out of hand is confirmed by the fact that others, as Shevrin and Luborsky (1961), and Steinig et al. (2017), have had the same bet. More fundamentally, even in the case that this type of rebus resolution would *not* happen, if such clinical observations as listed here are true, then the unconscious transformation of images to phonological traces should be possible (see also Shevrin and Fritzler, 1968). When two images are given side by side, if they are effectively phonologically translated and this phonological read-out has a form of independence to switch to other semantic realms, then this should be detectable in the slightly higher probability of rebus resolution. For this reason, the rebus paradigm, if it works, does suppose the phenomena which we want to show and contributes to the idea of a generally human rebus-mode of grasping the world.

There is, however, a big difference between the former dream-rebus research and the present one: we have not put ourselves in circumstances in which an increased unconscious functioning is supposed nor induced—we have not worked with dreams, nor with subliminal presentations. Indeed, we presumed that rebus resolution should not only be measurable in subliminal paradigms or when awakened during dreaming but also—though probably only very slightly (see further)—in supraliminal conditions in fully awake nonclinical participants. Therefore, we designed a rebus paradigm consisting of 14 French rebuses, composed of two images depicting monosyllabic words of easily identifiable common objects, such as e.g., *panthère* /pãtεr/ “panther,” composed by the images of *paon* /pã/ “peacock” and of *terre* /tεr/ “earth.” We presented these rebus images in a priming paradigm, i.e., the images were followed by a word related to the rebus resolution, e.g., *félin* “feline”; this is the rebus resolution, and the target words are semantic neighbors (such as, in this case, “panther” and “feline”). The participants were asked to look at the images for four seconds and then to write down the six first associations to the prime word. However, they were not specifically told to do something with the images, let alone that the images were forming a wordplay. The same prime words were also preceded by an unrelated rebus formed by two images, with the same instruction for six associations, constituting the inter-subject control situation. If more rebus-resolutions were counted among the associations upon the prime word, when this was preceded by its rebus than when it was preceded by an unrelated rebus, an influence of the rebus upon the associations must be supposed.

This, then, is our hypothesis, namely that *a pictorial environment can prime its reading on a phonological level—and thus, specifically, that two depicted images can prime their rebus reading—in a situation where the images were not convened linguistically*. This kind of passive phonological priming by pictorial materials was studied before, although in substantially different paradigms. For example, Humphreys et al. (2010) asked participants to associate upon a target word (e.g., cobweb) that was presented together with an image depicting an object with common phonemes in its name (here: the image of a spoon for the resolution word “spider”); participants were instructed to ignore the picture. They found more and faster spider-associations when the image of a spoon was presented suggesting that the common phoneme in “spoon” and “spider” had a facilitation effect in the association upon the target word “cobweb.” Studying cognitive and perceptual processes, Chabal and Marian (2015) test how overlapping picture names can influence visual-search performance: concretely, these participants are preferentially drawn to look at an image of a *cloud* in a picture (among other competing images) when this was preceded by the passive viewing of the picture of a *clock*, the effect being mediated by the common phonemes in the names of both items, even if no language was convened. Their conclusion supports the idea of an “automatic language activation during visual processing” (p. 548) even when language is not introduced in the task. These results are in line with other studies investigating how phonologically related pictures can facilitate the spoken-word production in picture naming tasks. For example, participants were asked to name the line-drawing of a bed (in green) superposed with one of a bell (in red) in the phonologically related, or with one of a hat (in red) in the unrelated distractor condition; participants were faster to name the bed when superposed with the image of a bell (Morsella and Miozzo, 2002). Others (e.g., Navarrete and Costa, 2005; Huettig and McQueen, 2007; Roelofs, 2008) have replicated this paradigm, including with briefly flashed action scenes (e.g., of diving or *tauchen*) priming for the naming of action scenes with rhyming names (e.g., *rauchen*, to smoke; Zwitserlood et al., 2018). These studies show the “automatic” effect of the visual environment upon the mental activity of participants subjected to this environment. Nevertheless, only one of these studies involved the reemergence of the complete name of the depicted object in the subject’s speech with a radically different meaning: Meyer and Damian (2007) show that people are faster to name the image of e.g., a bat (baseball) when superposed by a drawing of a bat (animal). This study is the closest to the clinical situation, where similar meaning reversals are observed (such as the squared *rand*-drawing stood for the *rand*-South African currency in the dream excerpt cited earlier). However, clinically, often the shifts in meaning are more spectacular with a tipping point similar in phonology even if quite dissimilar in orthography (such as *Käfer/Que faire*? and *comme il serre*/*commissaire*—see further) and probably therefore often unnoticed. Our research is especially interested in these kinds of shifts.

We have avoided the words “passive” or “automatic” to indicate the absence of the need to convene the images linguistically for there to be an effect. In a Freudian perspective, mental processing may go unnoticed by the subject himself without excluding subject specific influences; these processes are called “primary processes”. Indeed, the so-called primary process mental dynamic (Freud, 1900; see also Shevrin and Luborsky, 1961, p. 480) is an essentially associative mental dynamic, whereby connections are established based on so-called “superficial” (Freud, 1900, p. 597) or “non-essential” (Freud, 1905, p. 88; Holt, 1967, p. 354) characteristics, or even “attributes” (Rapaport, 1951, p. 708). Primary process mentation is thought to prevail in unconscious processing (Freud, 1915) but is also part of conscious mental dynamics. Primary processes function on a mirror-plane level with the stimulus in a direct action-reaction pattern, while for the secondary process to happen a third point, a perspective, is needed (Bazan, submitted). Secondary process mentation, to the contrary, keeps in mind the intentional structure of human reality and by doing so, can inhibit primary process connectivity (Freud, 1895, p. 334). For this reason, we will prefer the expressions “primary process” or “inadvertent” rebus priming, i.e., not achieved through deliberate planning. Moreover, Freud (1915, p. 186) said “By the process of displacement one idea may surrender to another its whole quota of cathexis; by the process of condensation, it may appropriate the whole cathexis of several other ideas. I have proposed to regard these two processes as distinguishing marks of the so-called primary psychical process”. As rebus resolution supposes the displacement of the cathexis to an attribute of the depicted object—its name—as well as its condensation with another name, rebus resolution without conscious injunction to resolve a rebus, is thought of as a primary process by excellence. Specifically, Shevrin et al. (1996, p. 107) claim that rebuses investigate “a formal aspect of dynamic unconscious thought organization marked primarily by superficial associations in the form of phonetic transitions and combinations,” which characterizes the primary process (Steinig et al., 2017). Importantly, primary process mentation is not concerned with spatial orientation: indeed, it is characterized by spatiotemporal confusion (Freud, 1900, 1936; Lacan, 1964, p. 40; Kapsambelis, 2005, p. 61) while spatiotemporal distinctions are, on the other hand, typical for secondary process mentation (Bazan, 2007). If rebus resolution is thought to happen along primary process logics, then people should resolve them as easily when the images are given in a forward order (*paon* /pã/ “peacock” + *terre* /tεr/ “earth”) and when they are given in the reverse order (*terre* /tεr/ “earth” + *paon* /pã/ “peacock”). Therefore, we included both a forward and a reverse condition in an intersubject set-up in Experiment 1. We could not risk intrasubject testing, as people are more easily inclined to consciously understand the rebus principle in the forward than in the reverse condition, and therefore the forward condition would have “contaminated” the reverse condition.

We remained with one concern, even if not a prohibitive one. Indeed, if priming by one image forming the rebus was also resulting in significant rebus resolution, then it remains in this set-up impossible to hold that the rebus solutions were to be explained obligatorily by the simultaneous depicting of two images and the subsequent condensation of their two names. Still, in that case, positive rebus resolution results would have corroborated our first hypothesis of a mental influence of the linguistically translated environment. But only if we would be able to show significantly *more* rebus resolution when prime words were preceded by the two images forming the rebus, and significantly less (or else, *no* significant) rebus resolution when the prime word was preceded by only one image, could we suppose that there indeed was inadvertent *rebus* priming. In our theoretical psychoanalytical framework, which implies supposedly complex mental unconscious processing, it is more convincing of the unsuspected capacities of the unconscious to show rebus resolution than to show ‘simple’ phonological priming by pictorial material. For this reason, we also included a second experiment, Experiment 2, in our research, implying the priming by only one of the two images, either the first or the last, in our set-up in an intersubject-configuration—as, again, in an intrasubject configuration with both one and two-image presentations, the complete rebus trials would have contaminated the one-image trials by enhancing insight into the principles underlying the protocol. Only if there is less, or else no significant, rebus resolution in Experiment 2 (one image) as compared to Experiment 1 (two images) our hypothesis of unwitting *rebus* resolution can be confirmed.

However, showing one-image phonological influence would still confirm the possibility of phonological priming by pictorial stimulus material. Phonological priming by linguistic stimulus material is, of course, a well-known effect very largely used to study naming processes (Meyer et al., 1974; Emmorey, 2002). However, this is explicitly not the present paradigm, which works with pictorial priming material, interrogating its phonological, not semantic, influence. But, of course, a conscious effort to retrieve the names of the presented pictures would transform the experiment into a “classical” case of phonological priming. Therefore, to avoid indirect phonological priming by linguistic primes, or, in psychoanalytic terms, to remain in a primary process mental logic, it is important to exclude the rebus resolution in participants who understood the rebus principle at some point during the research. Indeed, in that case, a conscious or attention-requiring effort to retrieve the names of the presented pictures may not be excluded. However, as will be shown, several people did indeed understand that the images were forming rebuses. For this reason, we had a thorough debriefing on people’s naivety at the end of the research and included only naive participants in all our analyses.

In sum, what we propose is that in any encounter of a subject with the visual world there will be a very marginal, though significant, rebus effect of this visual world upon the mental life of this subject. However, we only expect minimal rebus-effect as the mental influence of pictorial material is expected to be quite overwhelmingly visual (e.g., Kosslyn, 1994). Our proposition is that next to this well-known cognitive visual-semantic main effect, images can also prime for a different semantic realm than by the directly depicted one (the visuo-semantic translation) and this by way of their phonological translation. Nevertheless, if we want to make sense of the existing many clinical observations, this small effect should be significantly measurable even in these supraliminal conditions. Therefore, the present research thus proposes to verify if surrounding images, independently of their spatial configuration, have an inadvertent influence upon our mental productions through the phonology of their names and, moreover, that people produce rebus associations, independent of the order of image presentation, and significantly more so than by phonological association upon one image only. In other words, we propose to show that people solve rebuses unwittingly.

## 2. Experiment 1: Rebus priming

### 2.1. Materials and methods

#### 2.1.1. Participants

Participants were recruited on social networks, and all were majors. A total of 906 participants completed the entire online survey hosted by the Limesurvey platform (Limesurvey, 2020). Of the 906 participants, 118 participants were eliminated. First, 43 answered “yes” or “don’t know/don’t remember” to the question: “Do you remember having formerly participated in a study by Giulia Olyff?” and were consequently discarded. This question was asked at the beginning and at the end we reiterated the question “Do you remember having participated in a similar study?”. There were 15 pilot studies before this one, and it was important to avoid participants who were already exposed to rebus stimuli and might therefore be less naive to the rebus principle. Second, 75 participants were eliminated because they were not native French speakers or because they had a self-assessment of French language proficiency ≤ 5 on a 7-points Likert scale. French language proficiency was tested by an objective measure on 12 points, the FR12, which is a quick French test, which we designed for this study (see Supplementary material 1). After informed consent, our participants were randomly assigned to one of the two variations of the protocol (see further section “2.1.3 Conditions, variations, and randomization”). All demographic data are available in Table 1.

**TABLE 1:** Demographic data: Means (*M*) and standard deviations (SD) for *N* = 788 participants of Experiment 1.

	*M*(SD)
Age (years)	31.2(12.5)
Education (years)	15.5(1.8)
Self-assessment French proficiency (/7)	6.9(0.1)
French proficiency test (/12)	11.0(1.1)
Gender (% women)	76.6
Handedness (% right-handed)	84.5

#### 2.1.2. Stimuli

**FIGURE 2:**
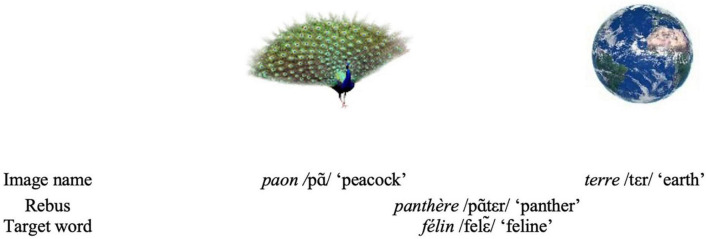
Example of an experimental stimulus composed of the target word *félin* “feline” and the images of *paon* /pã/ “peacock” and the image of *terre* /tεr/ “earth”; composing the French rebus *panthère* /pãtεr/ “panther”.

A stimulus is composed of a target word preceded by two images, forming a rebus. We designed 21 French rebuses composed by two images side by side. Each image represents a French monosyllabic word. Fourteen rebuses have an associated target word and together they compose our 14 stimuli. The remaining 7 rebuses that have no target word associated are our “supplementary rebuses” and are used for the CR condition (see further). For an example, see Figure 2, for the full list of stimuli see Supplementary material 2.

A total of 15 pilot studies, involving 3,327 participants, were conducted to develop our rebus priming method, including the selection of the rebuses, the rebus images, and the target words. To design our rebuses, we choose disyllabic French words of which the phonological translation can be divided into two monosyllabic French words. As a first requirement, the phonology of these words (forming the rebus) and the rebus in and by itself had to be perfectly identical. For example, *phobie* /fobi/ “phobia,” can be a rebus composed by the images of a *faux* /fo/ “scythe” and of a *bille* /bij/ “ball” but the match of the phonological transcription of the two images and the rebus resolution word was not perfect and the rebus was discarded (/fobi/ and /fobij/). Second, the imaged words and their rebuses must have (radically) different etymologies. For example, the rebus word *muraille* /myraj/ “wall” may not be formed by images of *mur* /myr/ “wall” and *ail* /aj/ “garlic,” as the etymology of *muraille* refers to *mur*. Therefore, while being homophonic, all the imaged words and the syllables of their rebuses were nevertheless orthographically dissimilar. In the cited example, we used the images of *mûre* /myr/ “blackberry” and *ail* /aj/ “garlic.” As a third requirement for the rebuses, they had to be composed by imageable, easy-to-name rebus components. For example, *baleine* /balèn/ “whale,” is a great rebus, formed by *bas* /ba/ “stocking,” and *laine* /lèn/ “wool,” but both images are hard to picture in a recognizable way (stockings are difficult to picture in an unambiguous way, it turned out we had a lot of confusion between stockings, pantyhose, and socks).

For the images, we kept colored pictures with no background chosen among copyright-free images on the internet with the best naming percentages (correct naming: *M* = 85.2%, *SD* = 10.56; for the list of images see Supplementary material 2).

For the target words, we use semantic neighbors. We selected words that were chosen to be at maximum slightly associated to the rebus. Indeed, highly associated target words would give high “rebus resolution” even in the control condition. For an objective measure of this association, we produced associative French norms to our target words (Olyff et al., submitted). The associative strength range between the target word and the rebus resolution was between 0 and 8.7%. These percentages indicate that, for example, when we presented the word *fromage* “cheese,” 4% of participants give as *first* association the word *dessert* in no-priming set-ups. The associative strength percentages predominantly serve to avoid highly associated target words. Even though the associative strength may be 0% for the first association, words are nevertheless chosen in association to the target in further associations. For our 14 stimuli the percentage associative strength between the target word and the rebus resolution word had a *M* = 1.8, *SD* = 2.73; for the list of associative strength between rebus resolution word and target word see Supplementary material 2.

#### 2.1.3. Conditions, variations, and randomization

Each target word could be presented in two conditions: the experimental condition (EX) or the control condition (CR). The target words in the CR condition must control for the spontaneous priming of the rebus resolutions, even without the related rebus images and in this case, with unrelated control rebus images. For the control condition we used unrelated rebuses because we cannot use rebus images of the experimental condition. Indeed, preliminary studies have shown that pseudo-randomizing target words with rebuses from the pool of experimental rebus image couples to obtain the control condition, gives rise to unwanted late priming effects. Consider e.g., the following situation: the images *rape* /rap/ “grater” and *as* /as/ “ace” used as control rebus images are followed by an unrelated target word; somewhat later another control stimulus is followed by an unrelated target word *aigle* “eagle”; though the images and the target words are mismatched, the coincidental order might still enhance the probability to respond *rapace* /rapas/ “raptor” to *aigle* “eagle” because of the preceding rebus combination *rape + as* exerting a late priming effect (*rapace* “raptor”). To eliminate the possibility of this effect altogether, we created 7 supplementary rebuses (pairs of images) that we exclusively reserved for the control condition. The control condition was then constructed by randomizing target words over the 7 control rebuses.

In summary, we had a total of 14 target words, associated to 14 pairs of images composing their related rebuses; these are experimental stimuli. Each participant associated upon the 14 target words. 7 targets words were presented in the EX condition, preceded by their corresponding rebus images, and the remaining 7 target word were presented in the CR condition, preceded by rebus images coming from the supplementary pool of rebuses. For example, in the EX the target word (i.e., *félin* “feline”) is preceded by its associated rebus (in this case *panthère* /pãtεr/ “panther”) composed by the two images side by side (i.e., image of a *paon* /pã/ “peacock” and of *terre* /tεr/ “earth”). In the CR the target word (i.e., *félin* “feline”) is preceded by an unrelated rebus (for example, *souplesse* /suplεs/ “flexibility”) composed by the two images side by side (i.e., image of a *soupe* /sup/ “soup” and of a *laisse* /lεs/ “leash”). Note that the *souplesse* rebus is thus one of the 7 supplementary French rebuses, designed specifically for the control condition, and therefore without associated target word (see section “2.1.2 Stimuli”).

**FIGURE 3:**
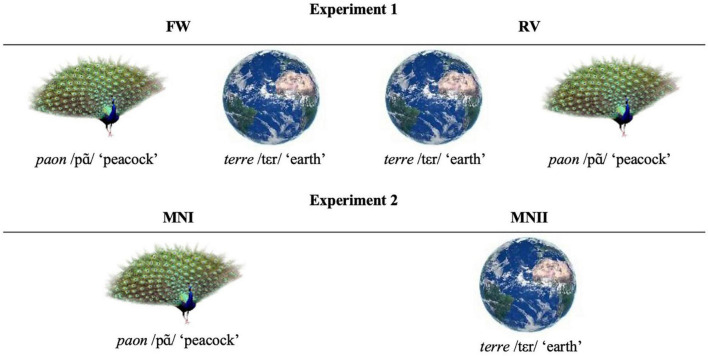
Variations for an experimental stimulus: *félin*—*paon terre*; composed by the images of *paon* /pã/ “peacock” and of *terre* /tεr/ “earth” giving the French rebus *panthère* /pãtεr/ “panther” and the target word associated to *panthère: félin* /felεϸ / “feline,” declined in the FW (forward) and RV (reverse) variations of Experiment 1 and in the MNI and MNII variations of Experiment 2.

Moreover, there were 2 variations for the presentation of our stimuli in Experiment 1: in the forward variation (FW) the images are presented in the forward order (i.e., the image of *paon* /pã/ “peacock” followed by the image of *terre* /tεr/ “earth”), and in the reverse variation (RV), in the *reverse* order (i.e., the image of *terre* /tεr/ “earth” and of *paon* /pã/ “peacock”). Participants were assigned either to the FW variation or the RV variation of Experiment 1 and received the 14 target words—as said, 7 of them in the EX condition (preceded by their related rebus) and 7 in the CR condition (preceded by an unrelated rebus; see Figure 3). It is important to note that this procedure implies that participants never saw twice the same couple of images nor twice the same target word.

#### 2.1.4. Funnel debriefing

To assess whether the participant stayed naive (or realized at one point) that the images presented side by side were forming a rebus, we composed an elaborate funnel debriefing, consisting of an 8-point evaluation. Note that the score is a *naivety score*: i.e., the higher the score, the naiver the participant. The structure of the debriefing is given in Table 2. Debriefings are adapted according to the variations (FW or RV) but follow the same logical development.

**TABLE 2:** Funnel debriefing in FW (forward) variation of Experiment 1.

Questions	Scores	
	*example*	w/o example*
1. Did you notice anything about the pairs of images that you have seen? If you can, give an example.	0	1 (correct elements)
2. Can you tell us something about these images?	2	3 (correct elements)
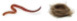		
3. The pairs of images you saw were *word plays*. If you can, give an example of such a word play. [*If an example of a correct rebus resolution is given:*] When did you understand this principle?**	4 (during the task) or 5 (after the task)	(go to 4)
4. The image pairs were word plays based on the names of the images. If you can, give an example.	6	(go to 5)
5. Take a good look at these two images. Can you solve this word play?	7	8 (no correct answer all over)
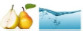		

For RV (reverse), all images were presented in reverse order. The rebus shown in question 2 is: *ver* /vèr/ “worm,” and *nid* /ni/ “nest,” forming *vernis* /vèrni/ “varnish”; the rebus shown in question 6 is: *poire* /pwar/ “pear,” and *eau* /o/ “water” forming *poireau* /pwaro/ “leek.” Note that as soon as either a correct rebus resolution or correct response elements are given, the scores are halted at the lowest naivety level.

*“example” means that participants receive the scores only if in the answer an example of a rebus resolution is given; “w/o example” means that participants receive the scores if correct elements of the rebus principle are given without giving an example.

**“during the task” was subdivided in: 1. since the first images seen; 2. during the task; 3. at the end of the task and 4. “after the task thanks to debriefing explanations.” “After the task” here refers to 4. “after the task thanks to debriefing explanations” option only.

As can be seen in Table 2, the highest weight is given to the ability to give a concrete example (of a formerly presented or of another rebus) or to solve a presented rebus. However, even if the participant can formulate an approximation of the rebus principle without giving an example in questions 1 or 2, he is considered non-naive. The pivotal point of the debriefing is on question 4, there we give away the information that the images are word plays. If the participant solves the rebus (only) at this point, he is considered non-naive, except in the case he indicates that he only just now came to understand the principle thanks to the debriefing information. In other words, participants are considered naive if the naivety score is ≥ 5 on 8.

#### 2.1.5. Procedure

The online survey started with written informed consent and inclusion criteria (older than 18 years and French speaking) followed by demographic and language questions (self-evaluation and FR12). Thereupon, participants received the following instruction (in French): “*Please put yourself in the best possible conditions to respond (far from distractions). In the next task, what interests us is your spontaneous associations based on the word that is presented to you, that is, the first thing that comes to mind when you read this word. Even if your answers seem absurd or trivial, follow your intuition. There are no right or wrong answers. What you are asked to do is: 1 - look at the images on the screen for 4 seconds. 2 - read the word that will follow 3 - write the first six words that come to mind associated to that word*.” The first stimulus appeared as two images side by side and stayed on screen for 4 seconds; then the stimulus as a target word appeared and participants received the following instruction: “*Please write the first six words that come to mind upon the word*: [target-word].” After the rebus task, participants answered the funnel debriefing.

#### 2.1.6. Rebus scoring and analysis

**FIGURE 4:**
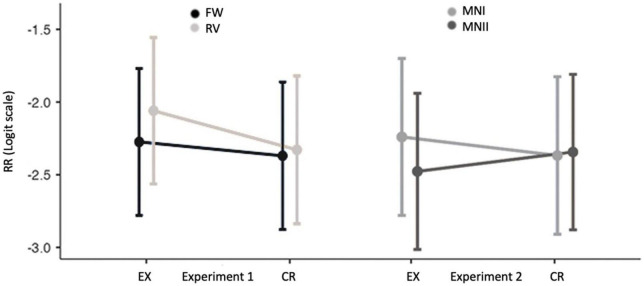
Fixed effects plots: linear predictor of the rebus resolution score (RR logit scale) for the condition experimental (EX) versus control (CR) and standard error for EX and CR conditions in the FW (forward) and RV (reverse) variations of Experiment 1 and EX and CR conditions in the MNI and MNII variations MNII (with only one of the two rebus images, respectively the first and the last) of Experiment 2.

We scored rebus resolution (RR) by attributing 1 point if the rebus resolution was found among the 6 written associations upon the related target word, independently of the fact if the rebus word appeared as the first or as a later association. This gives us a binary by-observation score (0 or 1). For example, for the target word *félin* “felin” we counted if *panthère* “panther” occurred among the 6 associations to *félin* “felin” either in EX or CR of both FW and RV. We analyzed the RR score using a Generalized Linear Mixed Model computed by the *GAMLj* module (Gallucci, 2019) of Jamovi software (2022) (R Core Team, 2021). We used Generalized Linear Mixed Models to determine the impact of the EX and CR conditions and of the FW and RV variations on rebus resolution for each observation. We wanted to know if the rebus resolution score depends on the target word being presented in the EX rather than in the CR condition and, simultaneously, if the order of the images (FW and RV variations) influences rebus resolution. We used the logit transformation for our analyses and included a by-subject and by-item intercept to consider differences among participants or items (in our case, target words), respectively. We added the naivety scores as well as the results on the FR12-test to control for the influence of naivety on rebus resolution, respectively of French language proficiency. We discarded model constructions with only either item or subject intercept and without Naivety Score or FR12 Score. In the results section, we present a model construction based on maximum likelihood and Akaike’s Information Criterion (Akaike, 1974; please refer to Supplementary material 3). To show our results in bar-plots (see Figure 4), we used percentual rebus resolution (%RR), by dividing RR for a target word by the total number of times the target word appeared in each condition of each variation. This gives us a percentage of rebus resolution per target word. To analyze the %RR we used a Wilcoxon Rank Test. We performed Bayesian paired t-Test (Rouder et al., 2009; Eidswick, 2012) in case of non-significant results to support the absence of effect (null hypothesis). For the BF_0+_ we used a Cauchy prior width of 0.707. Bayesian factor BF_0+_ quantifies for one-sided null hypothesis (Bayesian paired t-Test made by the *Jasp* module on Jamovi; JASP Team, 2018; Morey and Rouder, 2018).

### 2.2. Results

#### 2.2.1. Naivety results

On 788 participants that completed Experiment 1, 436 (i.e., 55.3 %) were naive to the rebus principle (see Table 3 for demographic data).

**TABLE 3:** Mean (*M*) and standard deviation (SD) of the demographic data; t- and p-results on the independent sample T-test between naive and non-naive participants; effect-size for the significant effect; two-tailed *p*-value.

	Naive *N* = 436	Non-naive *N* = 352			
	*M*(SD)	*M*(SD)	*t*	*p*	Effect size
Naivety score (/8)	7.0(1.0)	0.9 (1.2)	76.5	<0.00**	5.48
Mean number of rebus solved (/7)	1.0 (0.9)	3.2 (2.0)	20.2	<0.00**	-1.45
Age (years)	32.1 (13.0)	30.1(11.8)	2.23	0.02*	0.16
Education (years)	15.5(1.8)	15.4 (1.9)	0.16	0.8	
Self-assessment French proficiency (/7)	6.9 (0.1)	6.9 (0.1)	-0.6	0.4	
French proficiency test (/12)	11.0(1.2)	11.0 (1.0)	-0.5	0.6	

*p-value < 0.05; **p-value < 0.001.

There were no differences between the naive and non-naive participants on any of the demographic variables except for age. Naive participants are in average 2 years older that non-naive participants [*t*(786) = 2.23, *p* = 0.01, Cohen’s d = 0.16]. Nonetheless rebus solving was not correlated to age (*r* = −0.037; *p* = 0.29; *N* = 788). As expected, the Naivety Score of the naive population is much higher (7 times higher) than the score of the non-naive participants. Accordingly, the mean of rebuses solved (the number of rebus solutions in the 6 written associations on the 7 rebuses) of the EX condition was higher in the non-naive population compared to the naive [means of 3.2 and 0.96; *t*(786) = −20.2, *p* < 0.001, Cohen’s d = −1.45). In line with our hypotheses, we are exclusively interested in unwitting rebus resolution; therefore, we exclude the non-naive participants in further analyses. Interestingly, the Naivety Score is also significantly and substantially higher in the FW as compared to the RV variation [resp. 6.6(1.0) and 7.5(0.8); *t*(786) = 11.2, *p* < .001, Cohen’s *d* = 0.823].

#### 2.2.2. Do people solve rebuses unwittingly?

We describe here the Model with four fixed effects: Condition, Variation, Naivety score and FR12 as well as subjects and item (target words) as random effects; our target is the RR. Results are presented in Tables 4, 5 and Figure 4.

**TABLE 4:** Generalized linear mixed model for rebus resolution (RR) score in Experiment 1.

AIC	BIC	logLik		Deviance		def.resid	
4,202	4,256	−2,093		3,963		5,956	
	**Fixed effects**				**Random effects**		
					**By subject**		**By item**
**Effect**	**Estimate**	**SE**	***z***	***p***	**SD**		**SD**
(Intercept)	−2.188	0.415	−5.27	<0.001**	0.39		0.90
EX-CR	0.182	0.080	2.26	0.023*			
FW-RV	−0.127	0.097	−1.31	0.189			
EX-CR × FW-RV	−0.173	0.160	−1.07	0.281			
Naivety Score	0.009	0.047	−0.21	0.834			
FR12 Score	0.052	0.036	1.44	0.148			

Dependent variable is RR, the model family is binomial with a logit link. EX is the experimental condition; CR the control condition; FW the forward variation; RV the reverse variation, FR12 is the French proficiency test. Model formula: RebusScore ∼ 1 + Condition + Variation + Naivety + FR_12 + Condition:Variation + (1 | subjectID) + (1 | Target Word). *p-value < 0.05; **p-value < 0.001.

**TABLE 5:** Esteemed marginal means (*M)* and standard error (*SE*) for EX (experimental) and CR (control) rebus resolution (RR), as well as for EX and CR conditions in FW (forward) and RV (reverse) variations of Experiment 1.

Condition	Variation	*M*	*SE*
EX		0.1028	0.023
CR		0.0871	0.020
EX	FW	0.0933	0.021
CR	FW	0.0855	0.020
EX	RV	0.1131	0.025
CR	RV	0.0888	0.021

The analysis of the fixed effects (Table 4) shows a significant effect of the condition EX-CR on RR score (estimate 0.18, *p* = 0.02). The variation (FW-RV) value is non-significant (estimate −0.127, *p* = 0.19), the crossed effect between condition and variation is also non-significant (estimate −0.173, *p* = 0.28). Finally, the effects the Naivety Score and of the French proficiency (FR12) are non-significant (Naivety Score: estimate 0.00, *p* = 0.83; FR12: estimate 0.05, *p* = 0.14). The analysis of deviance using Type III Wald chi*-*square tests shows that Condition (related rebus presentation in experimental condition or unrelated in control condition) is a significant predictor of Rebus Resolution score χ^2^(df = 1) = 5.1408, *p* = 0.023, confirming the effect of EX versus CR condition. Variation (forward versus reverse order of images presentation) is not a significant predictor [χ^2^(df = 1) = 1.7220, *p* = 0.189], nor is the interaction between Condition and Variation variables [χ^2^(df = 1) = 1.1619, *p* = 0.281]. Also, Naivety Score and French proficiency score (FR12) are not significant predictors of the RR score [resp., χ^2^(df = 1) = 0.0440, *p* = 0.834; χ^2^(df = 1) = 2.0887, *p* = 0.148]. In other words, naive people solve rebuses unwittingly and this rebus resolution is not affected by the image order, the degree of naivety, nor by the French proficiency.

**FIGURE 5:**
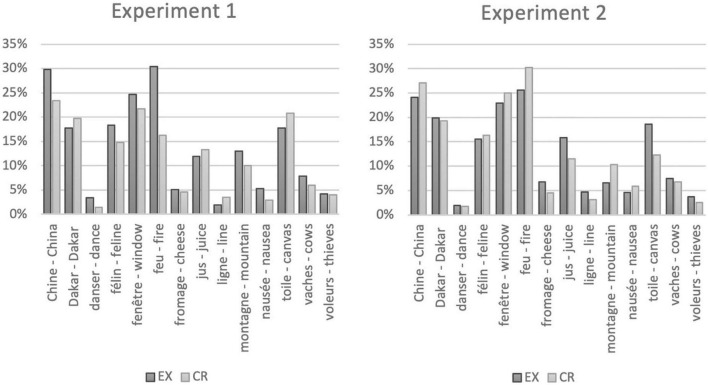
Bar-plot for the %RR (percentual rebus resolution) upon target word presentation in the EX (experimental—white bars) and CR (control—black bars) conditions of Experiment 1 and of Experiment 2.

We present in Table 6 %RR in EX and CR for each target word. Note that the rebus is identified by its related target word, and (counterintuitively) not by its composing images as, in the CR-condition of the same rebus, the composing images originally belong to a different rebus and only the target word is kept constant (and therefore images and target word are unrelated). The high variability in rebus resolutions upon the target words in the EX condition is paralleled by a high variability in rebus resolution in the CR condition, which is a good reassurance that the control condition indeed controls for the natural variability for the target words to spontaneously produce the rebus resolution in the EX condition. We present in Figure 5 bar-plots associated with %RR.

**TABLE 6:** Number of rebus resolutions, divided by the total number of times the target word appeared in each condition of each variation, presented as percentages (%RR), given by the naive participants to the target words in EX (experimental) and CR (control) conditions in, respectively, FW (forward) and RV (reverse) variations.

	FW		RV	
Target word	EX	CR	EX	CR
*Chine*—China	32.7	27.8	26.4	19.3
*Dakar*—Dakar	20.2	20.6	15.6	18.6
*danser*—dance	4.5	0.9	2.4	2.1
*félin*—feline	17.1	15.6	19.4	13.8
*fenêtre*—window	24.5	24.5	24.7	18.9
*feu*—fire	23.8	14.7	36.0	18.0
*fromage*—cheese	2.8	4.3	7.6	4.8
*jus*—juice	11.8	13.4	11.9	13.0
*ligne*—line	2.9	3.3	0.9	3.5
*montagne*—mountain	5.7	9.1	20.0	10.9
*nausée*—nausea	3.4	1.9	7.2	3.7
*toile*—canvas	19.1	18.0	16.0	23.2
*vaches*—cows	4.3	5.6	11.6	6.1
*voleurs*—thieves	2.7	2.7	5.8	5.2

Target words are presented in alphabetic order.

Results of the Wilcoxon test showed non-significant difference between the FW and RV variations for the mean EX %RR (FW *M* = 12.6, SD = 10.1; RV: *M* = 14.7, SD = 9.9; W = 33.0, *p* = 0.24, BF_0+_ = 1.8) nor for the CR %RR (FW *M* = 11.6, SD = 8.9; RV: *M* = 11.6, *SD* = 7.23; *Z* = 44.0, *p* = 0.63, BF_0+_ = 3.7). Between FW and RV for both EX and CR conditions BF_0+_ qualifies for moderate evidence in favor of null hypothesis (see Supplementary material 4 for the full detailed results). We further compared the %RR between EX and CR conditions and found, as expected, significant difference between EX and CR %RR in Experiment 1 (EX: *M* = 13.7, *SD* = 9.7; CR: *M* = 11.6, *SD* = 7.9; *Z* = 81.0, *p* = 0.03) with a medium effect-size (*r* = 0.54). In other words, there is inadvertent rebus priming by the visual environment of two images, independent of the order of these two images.

## 3. Experiment 2

### 3.1. Materials and methods

#### 3.1.1. Participants

A total of 550 participants completed the entire online survey hosted by Limesurvey (all were majors and recruited on social media platforms). 80 participants were eliminated: 34 because they already have participated to one of our pilot studies and 46 because they were not native French speakers or had insufficient self-assessment scores for French language proficiency (see section “2.1.1 Participants” for further details). Table 7 shows the demographic data for Experiment 2.

**TABLE 7:** Demographic data: Means (*M*) and standard deviations (SD) for *N* = 472 participants of Experiment 2.

	*M*(SD)
Age (years)	31.8 (12.8)
Education (years)	15.6(1.8)
Self-assessment French proficiency (/7)	6.9(0.1)
French proficiency test (/12)	11.1(1.1)
Gender (% women)	75.8
Handedness (% right-handed)	83.1

#### 3.1.2. Stimuli, variations, and randomization

The same stimuli of Experiment 1 were proposed in Experiment 2 but in their “mono” version: with only one out of the two images composing the rebus, either the first one (i.e., the image of *paon* /pã/ “peacock”; MNI variation) or the second one (i.e., the image of *terre* /tεr/ “earth”; MNII variation). In Experiment 2 we have followed strictly the same methodology as in Experiment 1: each participant was randomly assigned to either the MNI or the MNII variation and received 14 stimuli composed by one image and the target word (see Figure 3). The 14 stimuli were presented with 7 in the EX condition and 7 in the CR condition; for the CR condition, the 7 target words were randomly coupled with one image of a rebus coming from the supplementary control rebus pool, either the first image (MNI) or the last (MNII).

#### 3.1.3. Funnel debriefing

To assess whether the participant stayed naive (or realized at one point) that the image presented might have influenced participants associations in the form of word plays we adapted the funnel debriefing of Experiment 1 for Experiment 2. The structure and the scoring principle were identical for the two experiments (see Supplementary material 5 for the debriefing details of Experiment 2).

#### 3.1.4. Procedure

The procedure of Experiment 2 is identical to the procedure of Experiment 1. The only difference is the instructions that were adapted since participants saw only one image (and not two as in Experiment 1).

### 3.2. Results

#### 3.2.1. Naivety results

On 472 participants of Experiment 2, 469 (i.e., 99.3 %) were naive to the rebus principle (see Table 8 for demographic data); only 3 were non-naive.

**TABLE 8:** Mean (M) and standard deviation (SD) of the demographic data for Experiment 2; *t*- and *p*-results for the independent sample T-test between naive and non-naive participants; two-tailed *p*-values.

	Naive *N* = 469	Non-naive *N* = 3			
	*M*(SD)	*M*(SD)	*t*	*p*	Effect size
Naivety score (on 8)	7.8(0.3)	0.6(0.6)	40.9	<0.00**	23.3
Mean number of rebus solved (/7)	0.8(0.8)	2.6(2.3)	3.5	<0.00**	−2.07
Age (years)	31.9(12.7)	23.6(3.2)	1.1	0.2	–
Education (years)	15.5(1.8)	15.6(1.1)	−0.0	0.9	–
Self-assessment French proficiency (/7)	6.9(0.1)	7.0(0.0)	−0.2	0.8	–
French proficiency test (/12)	11.1(1.1)	11.5(0.7)	−0.4	0.6	–

**p-value < 0.001.

There were no significant demographic differences between naive and non-naive participant in Experiment 2. As expected, the Naivety Score of the naive population is much higher (more than 10 times higher) than the score of the non-naive participants. The mean number of rebuses solved by non-naive participants, resp. naive participants is 2.6 and 0.8 on a total of 7. The difference was significant [*t*(470) = −3.56, *p* < 0.001, Cohen’s d = −2.06]. In line with our hypotheses, we are exclusively interested in unwitting rebus resolution; therefore, we exclude the non-naive participants in further analyses. The Naivety Score is essentially identical in the MNI and MNII variations of Experiment 2 [resp. 7.9(0.2) and 7.8(0.3); *t*(470) = 0.44, *p* = 0.65].

#### 3.2.2. Inadvertent rebus priming by only one image?

We used the same model to analyze the unwitting effect of Condition (EX-CR) in the MNI and MNII variations of Experiment 2. Results are presented is Tables 9, 10 and Figure 4.

**TABLE 9:** Generalized linear mixed model for RR score in Experiment 2.

AIC	BIC	logLik		Deviance		def.resid	
4,365	4,419	−2,174		4,166		6,236	
	**Fixed effects**				**Random effects**		
					**By subject**		**By item**
**Effect**	**Estimate**	**SE**	***z***	***p***	**SD**		**SD**
(Intercept)	−4.876	1.232	−3.95	<0.001**	0.32		0.93
EX-CR	−0.002	0.078	−0.03	0.971			
MNI-MNII	0.105	0.085	1.23	0.216			
EX-CR * MNI-MNII	0.257	0.157	1.63	0.102			
Naivety Score	0.263	0.146	1.79	0.073			
FR12 Score	0.048	0.037	1.28	0.199			

Dependent variable is RR, the model family is binomial with a logit link. EX is the experimental condition; CR the control condition; MNI and MNII are the variations in Experiment 2 (with only one of the two rebus images, respectively, the first and the last), FR12 is the French proficiency test. Model formula: RebusScore ∼ 1 + Condition + Variation + Naivety + FR_12 + Condition:Variation + (1 | subjectID) + (1 | Target Word). **p-value < 0.001.

**TABLE 10:** Esteemed marginal means (*M*) and standard error (*SE*) for RR for EX (experimental) and CR (control) conditions in Experiment 2 and EX and CR conditions in MNI and MNII (with only one of the two rebus images, respectively the first and the last) variations of Experiment 2.

Condition	Variation	*M*	*SE*
EX		0.0857	0.020
CR		0.0860	0.020
EX	MNI	0.0954	0.023
CR	MNI	0.0850	0.021
EX	MNII	0.0770	0.019
CR	MNII	0.0869	0.021

The analysis of the fixed effects table (Table 9) shows non-significant effects of any of the variables of the model upon RR: Condition (estimate −0.00, *p* = 0.97), Variation (estimate 0.10, *p* = 0.21), interaction Condition*Variation (estimate 0.25, *p* = 0.10), Naivety Score (estimate 0.26, *p* = 0.07), the FR12 (estimate 0.04, *p* = 0.199). The analysis of deviance using Type III Wald chi*-*square tests showed that none of the following are a significant predictor of Rebus Resolution score in Experiment 2: Condition χ^2^(df = 1) = 0.0012 *p* = 0.971, Variation χ^2^(df = 1) = 1.5321, *p* = 0.216, the interaction between Condition and Variation χ^2^(df = 1) = 2.6704, *p* = 0.102; Naivety Score χ^2^(df = 1) = 3.2224, *p* = 0.073; French proficiency score (FR12) χ^2^(df = 1) = 1.6516, *p* = 0.199. In other words, people do not solve rebuses unwittingly with only one image. We present the %RR in EX and CR for each target word in experiment 2 in Supplementary material 6. Even if we also calculate the %RR here (see section 2.1.6 “Rebus scoring and analysis”), participants in this Experiment 2 did not see the two images composing the rebus but only one, either the first (MNI) or the second (MNII). In other words, we did count e.g., *panthère* “panther” responses in Experiment 2 even if the participant saw only the image of a “peacock” *paon* (MNI) or the image of the “earth” *terre* (MNII). We present in Figure 5 bar-plots associated with %RR for Experiment 1 and Experiment 2. Results of the Wilcoxon test showed non-significant difference between the MNI and MNII variations within the EX (MNI *M* = 13.9, *SD* = 9.9; MNII: *M* = 11.3, *SD* = 8.1; *W* = 67.0, *p* = 0.14, BF_0+_ = 1.5) nor for the CR condition (MNI *M* = 12.6, *SD* = 9.5; MNII: *M* = 12.5, *SD* = 10.4; *Z* = 55.0, *p* = 0.90, BF_0+_ = 3.7). Finally, we found no significant differences in %RR between EX and CR conditions (EX: *M* = 12.7, *SD* = 8.4; CR: *M* = 12.6, *SD* = 9.6; *Z* = 53.0, *p* = 0.64; BF_0+_ = 3.6) within Experiment 2. Bayesian factor BF_0+_ which quantifies for one-sided null hypothesis (i.e., EX %RR is not larger than CR %RR) qualifies moderate evidence in favor of null hypothesis (there is 3.6 more chances that EX %RR is not larger than CR %RR; see Supplementary material 7). In other words, there is no significant evidence that inadvertent rebus priming occurs also by only one image.

## 4. General discussion

Our results show that people solve rebuses unwittingly, with images presented both in forward and reverse order (Experiment 1), and that this rebus resolution is not the result of phonological priming by just one of both images (Experiment 2). Therefore, the rebus resolution must be the result of the simultaneous presentation of both images, and this in agreement with the fact that a rebus, indeed, implies the condensation of the two names of the images, following Freud’s (1900, p. 295) definition of condensation: “Condensation is brought about […] by latent elements which [are] fused into a single unity”. The rebus resolution is shown by our GLMM analysis which yields a significant effect of the difference between the experimental and the control conditions with an 0.182 estimate of the fixed coefficient (p = 0.023)^2^. The rebus influence, though statistically significant, seems very modest when verified by occurrence, but we did expect as much beforehand. Also, Zwitserlood et al. (2018, p. 17), for example, comment “as often the case in word naming [in phonological-priming-by images], the effect was numerically small,” in line with the findings of the present study. Nevertheless, the results of the statistical model tell us that the images have a small, though significant, rebus effect upon ensuing verbal association events when considered in an event-by-event manner.

Remarkably, the rebus resolution works as well both in forward and in backward (reverse) presentation of the two images. We did expect this result as we proposed that rebus resolution is carried by the Freudian primary process, which works independently of spatial configuration (for which, as a matter of fact, spatial configuration has no significance; Freud, 1900, 1936; Lacan, 1964, p. 40). This is also a very “ecological” result, in other words, resembling real-life situations, as in daily life images appear in very chaotic, unpredictable spatial configurations, but our results confirm this should not impede their “rebus impact” on mental life.

As a matter of fact, even if the differences are not statistically significant, the absolute numbers are in favor of the backwards (reverse) as compared to the forward image presentation. The one big difference between both conditions is that significantly more people acquire insight (called “perspicacity” henceforth, as the opposite of “naivety”) in the rebus principle in the forward as compared to the backward condition during the presentations (55.6% vs. 26.3% non-naive participants in the total populations for FW, resp. RV variations; see “2.2.1 Naivety results”). This might lead to the suspicion that there was nevertheless more perspicacity in the FW condition than in the RV even after selection of the non-naive participants with our debriefing filter. However, it must be stressed that the naivety factor is not significant in our GLMN analysis, disproving any residual effect of the naivety score on rebus resolution. Even when we verify this influence more specifically for the rebus resolution in the FW condition, we obtain *r* = 0.065; *p* = 0.34 between experimental rebus resolution and naivety scores (now see Supplementary material 8) which shows that there is none such correlation, nor a positive one, nor—as might have been presumed here, a negative one. Therefore, if we suspect a tendency of better rebus resolution in RV than in FW, this cannot be explained by a residue of conscious perspicacity with an inhibitory influence in FW, which might lead the participants to dismiss a seemingly irrational response at a conscious level: they would have insight but then would dismiss the rebus resolution, being blocked at the idea that e.g., a peacock and the earth are considered unrelated to a panther. However, in former research with the Shevrin lab we have found *unconscious inhibition* results: for example, in the so-called “pop look” studies of Snodgrass and Shevrin (2006), people who preferred to determine their identification response upon strictly subliminal stimuli by “looking” the best they could, gave *below-chance responses* as compared to people who claimed to be comfortable with letting responses spontaneously “pop up” (these latter people, gave above-chance correct identification responses). Similarly, in the Villa et al. (2006) palindrome study, supraliminal palindrome stimuli, such as e.g., “dog” (palindrome of “god”’) did not prime “angel” responses when given a forced choice between 2 conscious targets. However, when the palindrome prime (e.g., “dog”) was presented strictly subliminally, non-anxious people gave *significantly below-chance* correct responses (here “angel”) while highly anxious people gave significantly above chance correct responses. In a final study with reversible primes (e.g., “door”; Bazan et al., 2019), highly defensive participants chose the phonological inverse (here, “road”) *significantly less than chance* in a forced similarity choice (against an unrelated target, e.g., “lung”), while lowly defensive participants chose the phonological inverse at above chance levels. These various results show that at a subliminal, or unconscious level, inhibitory mechanisms start to come into play (see also Shevrin, 1992). It might be that this is also what we experience in the FW condition: one speculative explanation of our result tendencies might be that there is higher unconscious perspicacity in the FW than in the CR condition, in the same way that there is higher conscious perspicacity (filtered out by the debriefing). For a number of people this higher unconscious perspicacity might then induce unconscious dismissal of the rebus response (akin to a defensive or repressive response), explaining the response tendency in our research. However, this explanation remains at this stage speculative.

In the light of the former research on phonological priming by images (Morsella and Miozzo, 2002; Navarrete and Costa, 2005; Roelofs, 2008; Humphreys et al., 2010; McQueen and Huettig, 2014; Chabal and Marian, 2015; Zwitserlood et al., 2018), it is slightly suprising that we did not find significant rebus resolution when we only presented one image (Experiment 2). However, it should be noted that our measures were quite different from these in the cited studies, namely spontaneous associations (as in contrast to naming tasks) and that moreover, we did not count the occurrence of the single names (e.g., the number of *dés* “dice” or *serre* “greenhouse” for the *dessert* rebus) or the number of their direct derivatives such as (e.g., *dessert*—*délice*) in the associations; both of these aspects reducing substantially our probability to find phonological priming effects of the images. In other words, Experiment 2 shows that only one image is not enough for rebus priming but did not show the absence of phonological priming by one image.

However, our principal result, showing inadvertent rebus priming by the visual environment, indeed confirms, with a different paradigm, former phonological-priming-by-images research. More specifically, Morsella and Miozzo (2002, p. 561) having indeed found that superposed images help, by mere phonology of their names (i.e., not through their semantic meaning) the naming of other images, in their discussion wonder “whether phonology is always activated for all the things that happen to fall on the perceptual system, or whether this unintentional activation of phonology occurs only in the context of speech tasks.” With our results, and considering the clinical data, we are inclined to confirm the first idea, the irrepressible activation for all things “that happen to fall on the perceptual system,” even when language is not specifically convened.

One element that remains unclear is the need for attention to the presented images. Indeed Roelofs (2003, p. 366) proposes that “attentional enhancements are a precondition for obtaining phonological activation from pictures.” In our research we have indeed asked to pay attention to the images, but we have not otherwise implied them in the ensuing experimental task (the free association upon the target words); in other set-ups participants were not specifically called to pay attention to Morsella and Miozzo (2002), Meyer and Damian (2007), or were explicitly invited to ignore (Humphreys et al., 2010) the relevant images—nevertheless, in all cases, attention was indeed involved, which goes in the direction of Roelofs’ proposition. However, this question remains undecided as the phonological priming by images seems to work also in strictly subliminal conditions (Shevrin and Luborsky, 1961; Steinig et al., 2017). Moreover, a recent study of Chabal et al. (2022) shows that phonological priming of images occurs automatically and regardless of attention or memory demands.

Interestingly, Zwitserlood et al. (2018) show that the phonological-priming-by-images also works for action scenes, even when scenes do not have to be named—which widely opens up the naturalistic application of these findings to about anything present in the visual scene (and which is, by the way, also close to Freud’s idea, as his unresolved theoretical rebus example entails a *running* man, see above). This perspective upon larger implications as concerns the human mental world also resonates in Morsella and Miozzo’s (2002, p. 561) conclusions: “Beyond its implication for models of speech production, such a finding would bear on theories about the nature in which output programs are activated and selected in all actions, not just linguistic ones.” This track indeed leads to supposing structural linguistic influences not only in speech production, but “in all actions,” or even in all instances “in which output programs are activated,” i.e., more generally in the human behavioral and mental world—which would make sense with clinical observations.

We are hesitant to use the word “unconsciously” as our paradigm was a fully conscious, supraliminal paradigm and not a strictly subliminal paradigm, as was mobilized in the dream-rebus studies of Shevrin and Luborsky (1961) and Steinig et al. (2017). However, we had a strict debriefing procedure checking for the ability of the participant to report the word-play character of our images and selected only the naive participants in our analyses. As said, to completely exclude the possible contribution of any “perspicacity”-residues, which people wouldn’t have been able to report articulately, we also included “naivety” as a factor in our analyses and showed, by the absence of correlation that no rest of perspicacity influenced the results of our naive participants. In other words, we may be sure that the rebus resolution results found are not the result of a conscious insight into our rebus paradigm. Thereby, people can resolve rebuses without being able to report doing so, this is, if nothing more, *unwittingly*. Moreover, without this being proved by our results, we suggest that the processes underlying this ability are the processes proposed by Freud for the functioning of the unconscious, namely primary process mentation, as these processes are supposed to be active continuously in the “background” (Freud, 1901, p. 239) of conscious processing. This would be coherent of the reported effects being independent of the image order. Thereby, we consider our supraliminal paradigm as being in fact a very ecological one, close to “normal” mental life.

Finally, to exclude any biases of French proficiency upon our results, we strictly selected participants with a very high self-assessment of their French proficiency. For an independent check for this influence, we added a quick self-designed French test (“FR12”), which turned up to confirm that in our select group of participants French proficiency was not a significant predictor of our rebus effect.

Nevertheless, the ecological relevance of our findings might remain obscure as one might oppose: “We almost never encounter rebuses in real life”. Maybe there are more encounters than we think when a tie reaches a knee, and unconsciously hints to “tiny,” for example, but if we take the rebus-principle in a large, flexible way then we might find “ivan” in the divan or “Manhattan” when a man has his hat on, or we may feel primed to “arthrosis” when seeing a rose, to “carnival” upon seeing a car, to give a few possibilities. Opening this principle to action scenes, might imply that we are almost never unsolicited by slight phonological priming, as our attention is very often drawn to what is happening in the world. Even a marginal influence, integrated over so many visual takes a human being makes when being in the world, may create an accidental match between subject, situation, and environment, whereby the effect on human experience, is, we propose, a significant one. This is, it might help to explain why, for some subjects in specific situations, a black beetle elicits horror, the name of a shop induces well-being or the title of a book disgust, in a seemingly irrational, but nevertheless logically explainable way. Indeed, the relevance of these findings might be especially important in clinical situations: indeed, they might offer a rational ground for phenomena and their interpretation, which at first sight might seem completely “crazy”. For example, here is a remarkable excerpt of a patient with Tourette syndrome by the French neurologists Meige and Feindel (1902, p. 110) showing a conscious mobilization of the rebus principle: “to mimic the word commissary [*commissaire* in French] he [the patient] squeezed his right hand with his left hand [mimicking the comment] “how he squeezes!” [*comme il serre!* in French, phonologically close to *commissaire*] and to express doctor [*médecin* in French] he pretended to grasp on his chest imaginary breasts “my two breasts” [*mes deux seins* in French, phonologically close to *médecin*].”. Mostly the rebus structure of our “symptoms” remains obscure, even to ourselves, but in some personality structures—such as psychotic structures or Tourette syndrome—the unconscious is thought to be at the surface and rebus reading might be mobilized consciously. Our results contribute to the idea that the rebus principle is a general principle for mental functioning, and thereby helps to understand phenomena described here.

Another element coming from a different field also hints on the significance of rebuses for the human mind: indeed, their attested existence traces back to the beginnings of civilization. In the first writing attempts in history (such as e.g., in Sumerian and in Egyptian hieroglyphs, around 3000-3100 BC) images were used to fix language on support. A real writing revolution occurred when these “images” were used, not only for the representation of things but also for the representation of sounds—i.e., when humans invented the rebus principle. The statue of Ramsses II (dating around XIII century BC and conserved in the Museum of Egyptian Antiquities in Cairo), for example, shows a three part-composition depicting a solar disk, representing the sun God, called “Ra” in ancient Egyptian, a child called “mes” and a reed called “su” forming in fact Ra-mes-su or *Ramsses* : this is, Ramsses II’s name is on the statue in the form of a rebus, still accessible to deciphering after three thousand years. It is properly this *phoneme representation by images* called “phonogram” that in time leads to the inventions of alphabets and modern writing (Vernus, 2016, 2020). In other words, the rebus principle is a common principle to all writing systems and so much so that historians wonder if, to explain this universal phenomenon, we are not to suppose rebuses are a functioning principle of the human mind (Sington, 2020).

Given human’s apparently spontaneous appetite for and ease with such charades, it is slightly amazing that there aren’t more scientific studies on the topic. Among the few linguistic studies, most aren’t using our “picture + picture” principle but constitute miscellaneous combinations of words, letters, typographical writings, and pictures (see MacGregor and Cunningham, 2008; Salvi et al., 2015). As said, Freud is maybe among the first to give an explicit mental importance to rebuses, seeing them as the principle underlying dream formation, but also at the heart of “psychopathology of everyday life” such as in the forgetting of words (see the example of Signorelli, Freud, 1901). Therefore, it is in the psychoanalytic tradition, and specifically the tradition initiated by the American scientist and psychoanalyst, Howard Shevrin, that we find the most studies with direct relevance to the present one. Our research, even if it is directly inspired by Shevrin’s early rebus-dream studies, is, as said, however also different since, as a starter, it is a fully supraliminal paradigm. Moreover, Shevrin and colleagues did not use a priming set-up but asked for completely free associations (upon awakening participants in their REM-sleep). Therefore, given the discrepancies in experimental configurations, it is even more remarkable that the results are quite similar: people solve rebuses without knowing they do so, i.e., without any contribution of conscious cognitive computing, i.e., in an unconscious (Shevrin and Luborsky, 1961; Steinig et al., 2017), or at least, in an unwitting way (our study). For an exercise which at first sight seems cognitively complex and even remains challenging when doing it fully conscious of the nature of the materials, this is astonishing and contributes to the idea that there is a complex mental life with highly developed capacities, which is at work without us being aware of its influence. This was also part of Jacques Lacan’s amazement in his famous “return to Freud” (Lacan, 1955). With the results of this strictly experimental research, it is also our endeavor to, again, encourage such a linguistic “return to Freud,” as we think the linguistic model is the best model to grasp amazing clinical phenomena as given above, and, more fundamentally to grasp the irreducibly irrational foundation of mental functioning.

Our research also ambitions to bring a nuance to the prevailing visual-semantic paradigm to understand the human grasping of the world (e.g., Kosslyn, 1994). Next to grasping visual images in the world and being potentially submitted to their associative semantic activation in our mind, we propose that there is also an unnoticed, i.e. very discrete, *formal* influence of the phonology of the subtexts of our experience—the names and phrases catching what we live—upon our mental processing, but which, integrated over so many experiences, has a significant role, and may help to explain what we tend to call “subjective,” i.e., our “unexplainable” tastes and dislikes, obsessions and fears, and sometimes our mental symptoms.

Even if this study was preceded by a considerable number of preliminary studies (15; *N* = 3327 in total), which helped us to perfect our methodological protocol and our stimuli, we still have several considerations, which are important to include for a further perfectioning in case of replication of the protocol. First, as concerns the target words, the best rebus resolution results were found with the target words with an associative strength to the rebus resolution of 0%, which means that participants are almost never spontaneously giving the rebus resolution as a first associate to the target word. Indeed, the resolution appears in further associations, and this situation is the one, we suggest, giving the best sensitivity for the difference between the experimental and the control set-up. We suppose that with target-rebus resolution couples at 0% associative strength between both, our results would have been even stronger. Second, as discussed, there was a considerable variability among the rebuses. Among the limits of our research, we did not take into account the information on word frequencies (composing words, target words, rebus resolution words) which might help to explain this variability. However, the item variability was taken into account in the GLMM analysis. Third, as the experimental set-up seems to be quite sensitive to any “hints” giving away the rebus-principle, we suggest having non-rebus filler presentations in a systematic way—this is, presenting two images not forming a rebus and with no obvious relationship to the target word—to “drown” the rebus presentations. In a final consideration, with enough technical means, a *subliminal* (ideally, tachistoscopic) presentation of the rebus images, would of course be the litmus test to study unconscious processing.

In sum, our results with a supraliminal rebus paradigm show inadvertent rebus resolution of the visual environment as well as unwitting rebus resolution independently of image order, and show that this rebus solving is not due to the phonological priming by one of both images, but the result of a condensation of the names of both depicted objects. Our paradigm being an ecological one, this suggests that real life images appearing in chaotic spatial configurations have a discrete, formal influence upon our mental processing through the phonology of their related subtexts—the names and phrases associated to the experiences—which helps to explain subjective tastes and dislikes, and sometimes mental symptoms.

## Data availability statement

The raw data supporting the conclusions of this article will be made available by the author, without undue reservation.

## Ethics statement

The studies involving human participants were reviewed and approved by the Comité d’Avis Ethique de la Faculté des Sciences Psychologiques et de l’Education - Advisory Ethics Committe of the Faculty of Psychological Sciences and Education. The participants provided their written informed consent to participate in this study.

## Author contributions

Both authors listed have made a substantial, direct, and intellectual contribution to the work, and approved it for publication. Specifically, the experimental work was conducted by GO during the 6 years of her Ph.D. supervised by AB. This manuscript results from mutual support and the common work of authors that contributed equally to this article.

## Footnotes

1 or *rebus* and translated as “rebus” in the French translation. In the original German version: “*Ein solches Bilderrätsel ist nun der Traum*”.

2 Effect sizes are not generally produced in generalized linear mixed models (McCullagh and Nelder, 1989).
