# Supplementary material for: People solve rebuses unwittingly—Both forward and backward: Empirical evidence for the mental effectiveness of the signifier
Source: Front Hum Neurosci. 2023 Feb 10;16:965183. doi: 10.3389/fnhum.2022.965183 (PMC9951093; doi:10.3389/fnhum.2022.965183)
Supplement: Supplementary file 1 [file Data_Sheet_1.pdf]

Supplementary Material

1 The FR-12 test

Supplementary Table 1: FR12 test is loosely inspired by an online French School tests; the test is composed by 12 questions, grouped into 4 grammar questions (G); 4 vocabulary questions (V) and 4 comprehension question (C). Scoring was +1 for each correct answer. Correct answers are in bold.

	Question	Correct Response			
G	Penses-tu que Thomas viendra ce soir ? Il est peu probable qu'il ---.	**vienne**	viendra	vient	va venir
G	Je crains que vous --- trop nombreux pour tenir tous dans la même pièce	**ne soyez**	n'êtes	n'être	ne serez
G	Le bonheur est --- j'aspire dans la vie.	**ce à quoi**	ce dont	ce qui	ce que
G	Bien qu'il --- bien couvert, il a eu froid.	**se soit**	était	s'est	a été
V	Sa maison est au milieu d'une végétation --.	**luxuriante**	luxueuse	luxurieuse	enrichissante
V	Dépêche-toi, on va être à la ---- !	**bourre**	en avance	au beurre	en berne
V	Elle a une très belle voix mais elle ne chante pas --- du tout !	**juste**	vrai	faux	mieux
V	Le résultat de ces élections n'est pas représentatif à cause du taux massif ---.	**d'abstention**	d'absence	d'absentéisme	d'abstinents
C	Cet homme politique pratique la « langue de bois ». Cette expression signifie que son discours est agressif :	**faux**	vrai		
C	Ses arguments « me laissent de glace ». Cette expression signifie que ses arguments me laissent indifférent.e :	**vrai**	faux		
C	J'ai « la chair de poule ». Cette expression signifie que j'ai chaud :	**faux**	vrai		
C	Il a le bras long. Cette expression signifie qu'il est influent :	**vrai**	faux		

2 List of Stimuli

Supplementary Table 2: **List of Stimuli** presented as 1- Rebus: the rebus resolution word; 2- Images: the images composing the rebus resolution word; 3- Target Word; 4- the associative strength between rebus and target words (from Olyff et al., *submitted*). Note that norms refer to the first association upon the target word and in Experiment 1 and 2, participants gave their 6 firsts associations. For replication purposes, high definition images are avaible upon request from the corresponding author.

EXPERIMENTAL STIMULI

1- Rebus	2- Images	3- Target Word	4 – Associative Strength%
tourner /tuʁne/	tour /tuʁ/ - nez /ne/ 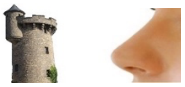	danser /dɑ̃se/	0%
vertige /vɛʁtiʒ/	vers /vɛʁ/- tige /tiʒ/ 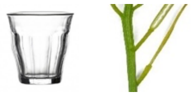	nausée /noze/	0%
pinceau /pɛ̃so/	pain /pɛ̃/ - seau /so/ 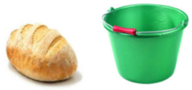	toile /twal/	1.0%
rallye /ʁali/	rat /ʁa/ - lit /li/ 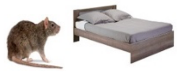	Dakar /dakar/	8.7%
métro /mɛtʁo/	mètre /mɛtʁ/- eau /o/ 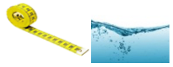	ligne /liɲ/	6.4%
pompier /pɔ̃pje/	pont /pɔ̃/ - pied /pje/ 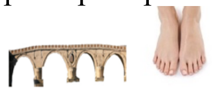	feu /fø/	1.9%
troupeau /tʁupo/	trou /tʁu/ - pot /po/ 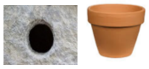	vaches /vaʃ/	0%
citron /sitʁɔ̃/	scie /si/- tronc /tʁɔ̃/ 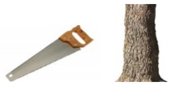	jus /ʒy/	3.8%
chalet /ʃalɛ/	chat /ʃa/ - lait /lɛ/ 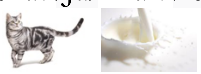	montagne /mɔ̃taɲ/	0%

1- Rebus	2- Images	3- Target Word	4 – Associative %
dessert /desɛʁ/	dé /de/- serre/sɛʁ/ 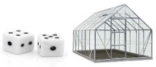	fromage /frɔmaʒ/	4.0%
rideaux /rido/	riz /ri/ – dos /do/ 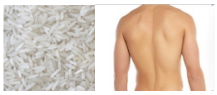	fenêtre /f(ə)nɛtr/	2.0%
muraille /myraj/	mûre /myr/– ail /aj/ 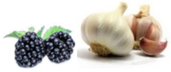	Chine /ʃin/	2.9%
braquage /brakaʒ/	bras /bra/ – cage /kaʒ/ 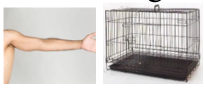	voleurs /vɔlœr/	0%
panthère /pɑ̃tɛr/	paon /pɑ̃/– terre /tɛr/ 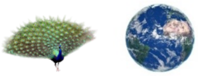	félin /felɛ̃/	1.8%

SUPPLEMENTARY REBUSES

coquille /kɔkij/	coq /kɔk/- quille /kij/ 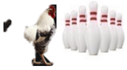
migraine /migrɛn/	mie /mi/- graine /grɛn/ 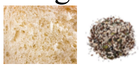
rapace /rapas/	râpe /rap/- as /as/ 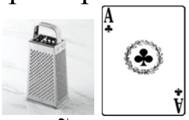
minceur /mɛ̃sœr/	main /mɛ̃/ - sœur /sœr/ 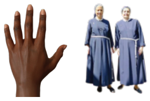
Platon /platɔ̃/	plat /pla/ – thon /tɔ̃/ 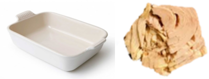
moustache /mustaʃ/	mousse /mus/ – tâche /taʃ/ 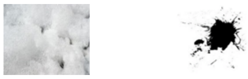
souplesse /suplɛs/	soupe /sup/ – laisse /lɛs/ 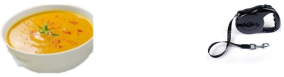

3 Models Comparaisons Experiment 1

	Model 1		Model 2		Model 3	
	β	SE	β	SE	β	SE
Intercept	-2,26	0,247**	-2,26	0,247**	-2,25	0,247**
EX – CR	0,202	0,07*	0,202	0,072*	0,182	0,080*
FW - RV	-0,108	0,08	-0,118	0,096	-0,119	0,089
EX-CR*FWRV	-0,216	0,15	-0,215	0,159	-0,173	0,160
Naivety Score			-0,013	0,46		
FR_12					0,053	0,036
Randomization Set						
Laterality						
Education						
Laterality*FW-RV						
**Random Effect variance**						
Participants	0,154		0,153		0,155	
Items	0,809		0,809		0,809	
**Model fit coefficient**						
AIC	4292		4294		4200	
BIC	4332		4341		4247	

Model 1 with no covariables formula : Score_binaire ~ 1 + EX-CR + FW-RV + EX-CR:FW-RV + (1 | Mot_cible) + (1 | ID)

Model 2 with no FR_12 score formula : Score_binaire ~ 1 + EX-CR + FW-RV + Naivety Score + EX-CR:FW-RV + (1 | Mot_cible) + (1 | ID)

Model 3 with no Naivety Score score formula : Score_binaire ~ 1 + EX-CR + FW-RV + FR_12 + EX-CR:FW-RV + (1 | Mot_cible) + (1 | ID)

	Model 4		Model 5		Model 6	
	β	SE	β	SE	β	SE
Intercept	-2,20	0,242**	-1,97	0,046**	-2,25	0,242**
EX – CR	0,177	0,080*	0,17	0,078*	0,183	0,080*
FW - RV	-0,124	0,088	-0,118	0,092	-0,129	0,088
EX-CR*FWRV	-0,174	0,160	-0,163	0,156	-0,172	0,160
Naivety Score	-0,009	0,042	-0,044	0,044	-0,005	0,042
FR_12	0,051	0,033	0,004	0,034	0,037	0,034
Randomization					0,006	0,005
Laterality					0,032	0,123
Education					0,023	0,024
Laterality*FW-RV					0,216	0,248
**Random Effect variance**						
Participants			0,107		0,151	
Items	0,790				0,810	
**Model fit coefficient**						
AIC	4210		4537		4215	
BIC	4256		4584		4289	

Model 4 with no subject intercept formula : Score_binaire ~ 1 + EX-CR + FW-RV + FR_12 + EX-CR:FW-RV + (1 | Mot_cible)

Model 5 with no item intercept formula : Score_binaire ~ 1 + EX-CR + FW-RV + FR_12 + EX-CR:FW-RV + (1 | ID)

In model 6 the factor 'Randomization' indicates that our Randomization was, in fact, not a real randomization but a rotation over 24 sets in which each of the 14 targets words were presented either in EX or in CR conditions. Every set was different, but we balanced the number of times each target words appears in EX or CR, so among the 24 sets each target word appears 12 times in EX and 12 times in CR condition.
In other words, we have a pool of 14 experimental stimuli, constituted by 14 couples of images and their belonging target words (see 3.1.2 'Stimuli'). Each participant receives 7 items in this experimental condition. The 7 remaining target words were randomly combined with 7 image couples of the pool of Supplementary Rebuses (see 3.1.2 'Stimuli' and 3.1.3 'Conditions, Variations and Randomization).
We were faced with the problem that this randomization could lead to unwanted combinations of "(supplementary) rebus images + target word" coincidentally acquiring a proper sense and thereby unlawfully influencing the priming results. For example, when the target-word *ligne* 'line' (belonging to the experimental rebus *metro*) is preceded by control rebus *souplesse* 'flexibility', *ligne* 'line' might be inclined to prime for *souplesse* 'flexibility' instead of *métro* 'subway' because of the semantic link (in French) between 'stay in shape' (*garder la ligne)* and 'flexible'. This would give unfairly low numbers of *métro* responses upon the control stimulus with the *ligne* target and therefore this combination was not allowed. To avoid these unwanted priming, we created 24 sets (experimental + control rebuses) in which these combinations were avoided and rotated them over the participants. Model 6 shows that this rotation had no influence whatsoever on these results.

Model 6 with all variables formula : Score_binaire ~ 1 + EX-CR + FW-RV + Naivety Score + FR_12 + Randomization Stets + Laterality + education + EX-CR:FW-RV + Laterality:FW-RV + (1 | Mot_cible) + (1 | ID)

4 Details for BF_{o+} Experiment 1

Supplementary Table 4: Bayesian Paired Samples T-Test for %RR (percentage Rebus Resolution) of EX (experimental) and CR (control) conditions between FW (Forward) and RV (Reverse) variations in Experiment 1 . *N* = 14 Target Words

		**BF_{01}**	**Error %**
FWEX	RVEX	1,78	0,01785
FWCR	RVCR	3,69	0,00911

Prior and Posterior distribution **BF Robustness Check**

FWEX - RVEX

FWCR - RVCR

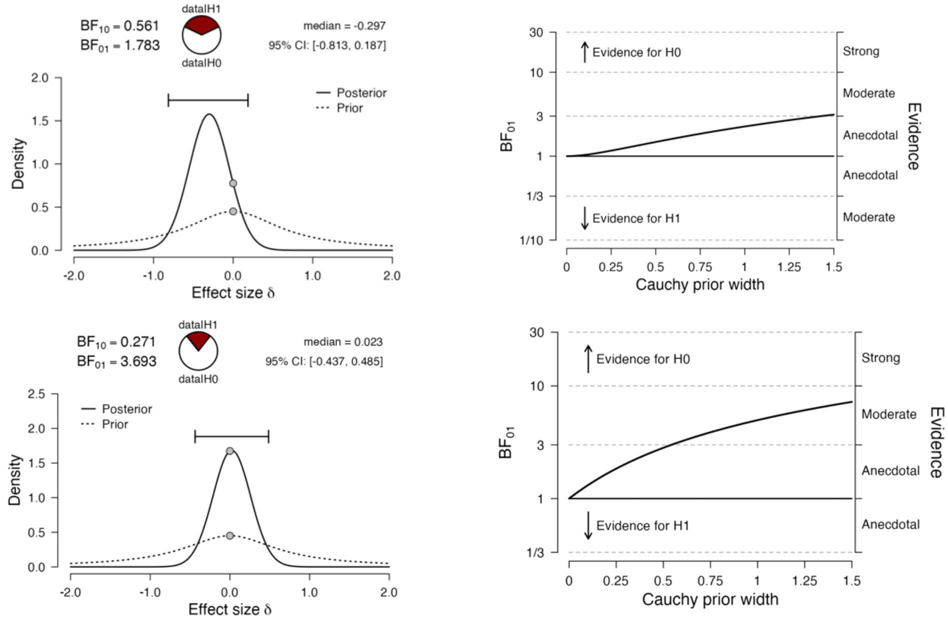

5 Funnel debriefing details for Experiment 2

Supplementary Table 3: Funnel debriefing in Experiment 2 where only one image was presented. The image shown in question 2 is: *ver* /vèr/ 'worm'; the image shown in question 6 is: *eau* /o/ 'water'. Note that as soon as a correct response element is given, the scores are halted at the lowest naivety level.

Questions	Score (/8)	
	example	*w/o example**
1. Did you notice anything about the images that you have seen? If you can, give an example.	0	1 (correct elements)
2. Can you tell us something about this image? 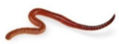	2	3 (correct elements)
3. **The images you saw might have influenced your associations in the form of a *word play*.** If you can, give an example of such an influence. [*If an example of a correct influence is given:*] When did you understand this principle? **	4 (during the task) or 5 (after the task)	(go to 4)
4. The image might have influenced your associations by ways of its name. If you can, give an example.	6	(go to 5)
5. Take a good look at this image. Can you solve this word play ? 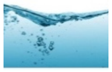	7	8 (no correct answer all over)

*'example' means that participants receive the scores only if in the answer a correct example is given; 'w/o example' means that participants receive the scores if correct elements of the priming principle are given without an example.
** 'during the task' was subdivided in: 1- since the first image seen; 2- during the task; 3- at the end of the task and 4- 'after the task thanks to debriefing explanations'. 'After the task' here refers to 4- 'after the task thanks to debriefing explanations' option only.

6 %RR for Experiment 2

Supplementary Table 6: Number of rebus resolutions, divided by the total number of times the target word appeared in each condition of each variation, presented as percentages (%RR), given by the naive participants to the target words in EX and CR conditions in, respectively, MNI and MNII variations. Target words are presented in alphabetic order.

	**Experiment 2**			
	**MNI**		**MNII**	
**Target Word**	**EX**	**CR**	**EX**	**CR**
Chine - China	31.9	32.0	31.9	32.0
Dakar - Dakar	23.5	19.1	23.5	19.1
danser - dance	2.3	2.1	2.3	2.1
félin - feline	15.3	16.3	15.3	16.3
fenêtre - window	18.1	24.1	18.1	24.1
feu - fire	30.1	24.5	30.1	24.5
fromage - cheese	11.2	5.6	11.2	5.6
jus - juice	13.3	12.8	13.3	12.8
ligne - line	4.7	2.0	4.7	2.0
montagne - mountain	9.8	7.5	9.8	7.5
nausée - nausea	4.7	5.9	4.7	5.9
toile - canvas	20.7	14.4	20.7	14.4
vaches - cows	4.0	4.9	4.0	4.9
voleurs - thieves	3.9	4.1	3.9	4.1

7 Details for BF_{0+} Experiment 2

Supplementary Table 7a: Bayesian Paired Samples T-Test for %RR (percentage Rebus Resolution) of EX (experimental) and CR (control) conditions between MNI and MNII variations in Experiment 2. *N* = 14 Target Words

		**BF_{01}**	**Error %**
EXMNI	EXMNII	1,53	0,019
CRMNI	CRMNII	3,70	0,009

Prior and Posterior distribution **BF Robustness Check**

EXMNI EXMNII

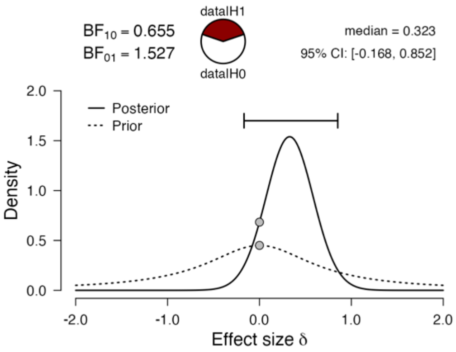

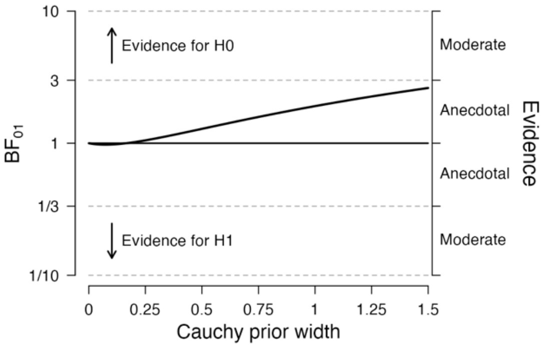

CRMNI CRMNI I

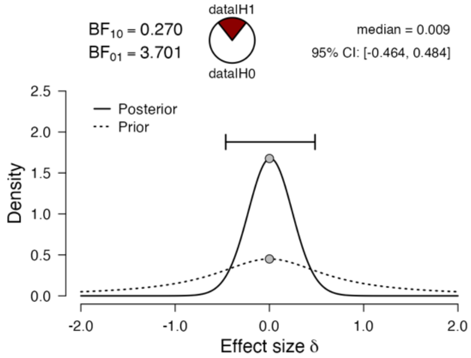

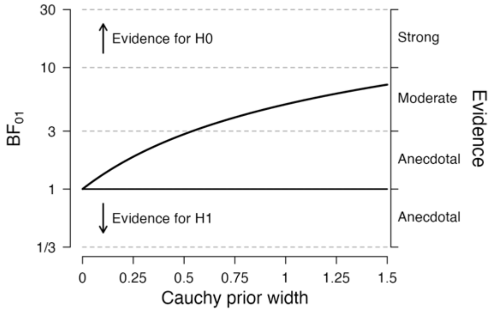

Supplementary Table 7b: Bayesian Paired Samples T-Test for %RR (percentage Rebus Resolution) of EX (experimental) and CR (control) conditions in Experiment 2. $N = 14$ Target Words

		**BF_{01}**	**Error %**
$EX_{Experiment2}$	$CR_{Experiment2}$	3,36	0,015

	**Prior and Posterior distribution**	**BF Robustness Check**
**EX-CR Experiment 2**	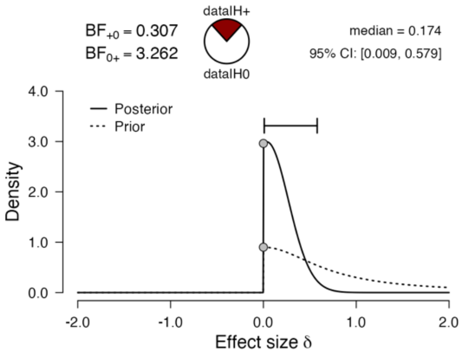	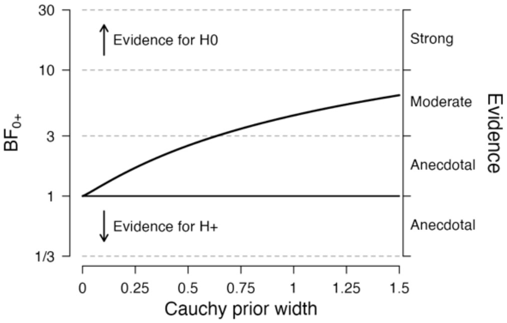

8 Details for correlations between number of rebuses solved and naivety

Supplementary Table 8: Correlations (Pearsons's ro) and Bayesian Correlation between the number of rebus resolutions in EX given by the naive participants to the target words, respectively, in FW and RV variations of Experiment 1.

	Naïvety Score		
**RPP**	**Experiment 1**	**Forward Variation**	**Reverse Variation**
r=	.027	.065	-.109
p=	.577	.335	.111
BF_{10}	.070	.134	.302

BF_{10}= Bayesian Factor in favor of alternative hypothesis: there is a correlation
*= $p<.05$ or $BF_{10} >10$; **= $p<.01$ or $BF_{10} >30$; ***= $p<.001$ or $BF_{10} >100$
